# *Medicago truncatula* Gaertn. as a model for understanding the mechanism of growth promotion by bacteria from rhizosphere and nodules of alfalfa

**DOI:** 10.1007/s00425-016-2469-7

**Published:** 2016-02-10

**Authors:** Anna Kisiel, Ewa Kępczyńska

**Affiliations:** Department of Plant Biotechnology, Faculty of Biology, University of Szczecin, Wąska 13, 71-415 Szczecin, Poland

**Keywords:** ACC deaminase, Bacteria identification, Growth promotion, Indole acetic acid (IAA) content, PGPR traits, *Phl*D gene detection

## Abstract

**The present study showed all the 16 strains isolated and identified from the alfalfa rhizosphere and nodules, and registered in GenBank, to be good candidates for targeted use in studies addressing the rather weak known mechanism of plant growth promotion, including that of*Medicago truncatula,*a molecular crop model.**

Based on physiological, biochemical and molecular analysis, the 16 isolates obtained were ascribed to the following five families: Bacillaceae, Rhizobiaceae, Xantomonadaceae, Enterobacteriaceae and Pseudomonadaceae, within which 9 genera and 16 species were identified. All these bacteria were found to significantly enhance fresh and dry weight of root, shoots and whole 5-week-old seedlings. The bacteria were capable of the in vitro use of tryptophan to produce indolic compounds at various concentrations. The ability of almost all the strains to enhance growth of seedlings and individual roots was positively correlated with the production of the indolic compounds (*r* = 0.69; *P* = 0.0001), but not with the 1-aminocyclopropane-1-carboxylate deaminase (ACCD) activity (no correlation). For some strains, it was difficult to conclude whether the growth promotion was related to the production of indolic compounds or to the ACCD activity. It is likely that promotion of *M. truncatula* root development involves also root interaction with pseudomonads, known to produce 2,4-diacetylphloroglucinol (DAPG), a secondary metabolite reported to alter the root architecture by interacting with an auxin-dependent signaling pathway. Inoculation of seedlings with *Pseudomonas brassicacearum* KK 5, a bacterium known for its lowest ability to produce indolic compounds, the highest ACCD activity and the presence of the *phl*D gene responsible for DAPG precursor synthesis, resulted in a substantial promotion of root development. Inoculation with the strain increased the endogenous IAA level in *M. truncatula* leaves after inoculation of 5-week-old seedlings. Three other strains examined in this study also increased the IAA level in the leaves upon inoculation. Moreover, several other factors such as mobilization of phosphorus and zinc to make them available to plants, iron sequestration by siderophore production and the ability to ammonia production also contributed substantially to the phytostimulatory biofertilizing potential of isolated strains. There is, thus, evidence that *Medicago truncatula* growth promotion by rhizobacteria involves more than one mechanism.

**Electronic supplementary material:**

The online version of this article (doi:10.1007/s00425-016-2469-7) contains supplementary material, which is available to authorized users.

## Introduction

The family Fabaceae, called also Leguminosae, is the third largest family among angiosperms and second only to the Gramineae in their importance for humans (Graham and Vance 2003). The family’s most commercially important species worldwide include the soybean (*Glycine max*), the garden pea (*Pisum sativum*), the peanut (*Arachis hypogaea*) and the alfalfa (*Medicago sativa*). Those plants are the most important source of protein and oil for humans and animals, and enrich the soil with nitrogen. They are also important as a fuelwood and with respect to carbon (C) sequestration (Abberton 2010). Among forage crops, cultivation of *M. sativa* covers the largest acreage worldwide and produces the largest profit. Furthermore, the alfalfa, one of the most important small legumes, is highly adaptable to different climatic and soil conditions, which facilitate its cultivation worldwide. Cultivation of alfalfa has, for many years, been known to bring benefits for the soil, e.g., structure improvement, protection against wind and water erosion, nitrogen and organic enrichment, and weed population reduction. The increasing concerns about a decline in soil organic matter content and fertility as well as the rising costs of energy and nitrogen fertilizers have renewed the interest in legumes.

Recently, the role of legumes and their contribution to both the sustainable intensification of agricultural production and the livelihoods of small-holder organic farmers in many parts of the world have featured on the research and economic agendas. Alfalfa is grown for hay, pasture, seed and—in some areas—as a major crop for the dehydration industry. It also finds its use in many medical formulae, and the available preparations such as alfalfa tonic and malt are known for their health benefits on account of a variety of bioactive natural products the alfalfa-based preparations contain (Gholami et al. 2014). The seed production in the temperate climate is limited mainly by the too short growing season due to the low temperature and insufficient sunlight resulting in delay of the flowering time. Healthy and well-rooted plants obtained in a relatively short time ensures their withstanding adverse conditions and the possibility of the perennial alfalfa producing seeds in the year of sowing. The measures to improve plant quality and health include the use of synthetic fertilizers and pesticides. In the context of organic farming, however, it is mandatory to look for other, more efficient and also environmentally safe methods to improve germination, seedling emergence and plant health. One such method can be the use of rhizosphere microorganisms, including the plant growth—promoting rhizobacteria (PGPR) (Chandler et al. 2008; Glick 2012). Numerous authors have reported application of PGPR, via seed coating or as inoculants after seedlings have emerged, to improve seedling growth of many plants (Khan et al. 1990; Glick 1995; Gholami et al. 2009; Bhattacharyya and Jha 2012). PGPR used include genera such as *Bacillus*, *Pseudomonas*, *Erwinia*, *Serratia*, *Arthrobacter*, *Micrococcus*, *Flavobacterium*, *Azotobacter*, *Azospirillum*, *Rhizobium* and *Agrobacterium* (Verma et al. 2010; Glick 2012). Although the mechanisms used by PGPR to directly or indirectly promote plant growth are not fully understood yet, several have been suggested. Based on their mode of action, PGPR are classified as biofertilizers, phytostimulants and biopesticides, although many PGPR often use more than one mode of plant growth enhancement (Bhattacharyya and Jha 2012; Glick 2012). A direct phytostimulation may include the production of phytohormones, e.g., gibberellins, cytokinins and auxins as well as modulation of ethylene level, a gaseous phytohormone, by bacterial ACC deaminase (ACCD) or enhanced provision of nutrients. Indole acetic acid (IAA) produced by bacteria colonizing the plant rhizosphere is considered to act in conjunction with the endogenous plant IAA to stimulate root proliferation and elongation resulting in a more branched root system architecture, which enhances the host’s uptake of minerals and nutrients from the soil (Mantelin and Touraine 2004; Mantelin et al. 2006; Spaepen et al. 2007; Shokri and Emtiazi 2010). However, PGPR which do not produce auxin are known to be able to modify the endogenous transport of plant IAA or to regulate auxin homeostasis by, e.g., production of volatile organic compounds (VOC), resulting in the same root architecture effects (Zhang et al. 2007; Contesto et al. 2010; Zamioudis et al. 2013). Some PGPR belonging to fluorescent pseudomonads are well-known producers of the antimicrobial compound 2,4-diacethylphloroglucinol (DAPG) which, at a low concentration, can enhance root branching (Brazelton et al. 2008; Couillerot et al. 2011).

One of the most important factors in seeking an increase of the PGPR efficacy is to find the best bacteria available. Bhattacharyya and Jha (2012) listed the commercially available products used worldwide in plant (mainly cereal) growth promotion, but the list seems to be too short. This is due, i.a., to the fact that neither all the mechanisms the bacteria use for promoting plant growth have been fully elucidated, nor the plant response mechanisms during PGPR treatment are completely known (Vacheron et al. 2013).

Thus, it is essential to explain these mechanisms at various levels, including molecular. Such studies should avoid using *Arabidopsis thaliana*, a weed serving to date as a molecular model, but should instead use a crop plant, *Medicago truncatula* Gaertn. known as the barrel medic. In recent years has this plant gained great interest; its attractiveness compared with alfalfa (*M. sativa*) is related to its more rapid vegetative growth (12–16 weeks from seed to seed) and a high seed production, associated with low requirements for light, temperature, and substrate composition. In contrast to the alfalfa, it does not require pollinators such as bees (Cook 1999; Rose 2008). Moreover, *M. truncatula* belongs to the phylogenetic group which also includes alfalfa, peas, beans, chickpeas and clover. It can, thus serve as a model plant because it has a small diploid genome which is already known (alfalfa is tetraploid). Furthermore, genetic mapping of *M. truncatula* and *M. sativa* showed a high level of synteny. This fact will allow future use of genetic and genomic tools, created on the basis of *M.truncatula*, to improve productivity and cultivation of alfalfa by, i.a., understanding of plant growth promotion mechanisms by PGPR. It is a model plant for molecular and genetic studies of legumes mainly because of its symbiotic association with rhizobia, but also on account of its interaction with other non-symbiotic growth-promoting bacteria (PGPR), arbuscular mycorrhiza (AM) fungi or pests and pathogens (Barker et al. 1990; Hohnjec et al. 2006; Rose 2008).

To date, no reports on the application of rhizobacteria to promote the development of *M. truncatula* have been published, and no data on bacteria species identified from the *M. sativa* rhizosphere. Therefore, the work reported here was aimed at: (1) isolating bacteria from the rhizosphere of the legume *M. sativa* L.; (2) evaluating their physiological and genetic characteristics; (3) screening them for their promoting capacities with respect to *M. truncatula* Gaertn., and finally evaluating their application as environmentally friendly adjuncts to agriculture practice—as biostimulants and biofertilizers.

## Materials and methods

### Isolation of rhizobacteria

Strains of bacteria were isolated from the rhizosphere and nodules of *M. sativa* L. from the region of Western Pomerania in Poland. The *Medicago* roots were washed with 5 ml of sterile distilled water and the soil solution, thus, obtained was used for isolation of non-symbiotic bacteria using the method of Penrose and Glick (2003) with some modifications. The root nodules were separated from the roots and sterilized in 95 % ethanol for 5 min; they were then rinsed with sterile water and homogenized using a micro-pestle. The root nodule homogenate was used for isolation of symbiotic bacteria. The soil solution and the nodule homogenate were placed in 20 ml of Tryptic Soy Broth (TSB) and 2xYT (Liquid Yeast Tryptic) medium, respectively, in 50-ml Falcon tubes. The suspensions were incubated in a shaker incubator (200 rpm) at 28 °C. After 24 h, a 1 ml of the growing culture was transferred to 20 ml of TSB or 2xYT medium and incubated for another 24 h. Following the two incubations, 1 ml of both suspensions each was transferred to 20 ml of minimal medium and DF salts (Dworkin and Foster 1958), respectively, supplemented with ammonium sulfate or M9 salts supplemented with ammonium chloride as a nitrogen source. After incubation, a 1 ml aliquot was removed from the culture and transferred to 20 ml DF salts or M9 salts with 5 mM ACC as a source of nitrogen, and was grown for 24 h under conditions identical as those used in the first incubation. A 100 µl portion of the final culture was plated onto solid DF or M9 salts minimal medium containing 5 mM ACC and 2 % Bacto-Agar, and was incubated for 72 h at 28 °C. Morphologically different colonies appearing on the medium were isolated and purified by streaking on plates with the same medium, placed in cryovials (Scharlau Cryoinstant) and kept at −80 °C for further characterization and identification.

### Phenotypic characterization of bacterial isolates

Physiological and biochemical characteristics of the bacterial isolates were examined according to the methods described in Bergey’s Manual of Determinative Bacteriology (Holt et al. 1994). The bacterial isolates were grown on Tryptic Soy Agar (TSA) at 28 °C for 24 h. Phenotypic characterization of the isolates was conducted based on their colony morphology. Subsequently, the cellular morphology of pure cultures of the isolate (after Gram staining) was analyzed by light microscopy. PGPR motility was determined and some standard biochemical tests (starch hydrolysis, lactose fermentation, resistance to streptomycin and ability to fluorescence) were applied following methods described in Bergey’s Manual of Determinative Bacteriology (Holt et al. 1994).

#### PCR amplification and sequencing

All the strains isolated were characterized based on 16S ribosomal RNA (rRNA) gene sequences and additionally on the *rpoD* (coding RNA polymerase sigma factor) and *gyrB* (coding gyrase B) genes of the strains assigned to the genus *Pseudomonas* (Cladera et al. 2004). The bacteria were cultured in TSB or 2xYT medium at 28 °C (the shaker set at 200 rpm). A 1-ml portion of the cell suspension was centrifuged at 5000*g* for 10 min. The cell pellets were resuspended in 300 µl lyse buffer and used to purify the genomic DNA using the DNA Extraction Kit (Eurx, Gdansk, Poland). The following primers were used for the polymerase chain reaction (PCR) amplification of the 16S ribosomal RNA: 16S rRNA-F (5′-AGCGGCGGACGGGTGAGTAATG-3′) and 16S rRNA-R (5′-AAGGAGGGGATCCAGCCGCA-3′) (Young et al. 2004) and UP-1E (5′-CAGGAAACAGCTATGACCAYGSNGGNGGNAARTTYRA-3′) and APrU (5′-TGTAAAACGACGGCCAGTGCNGGRTCYTTYTCYTGRCA-3′) for *gyrB* gene amplification and 70Fs (5′-ACGACTGACCCGGTACGCATGTA-3′) and 70Rs (5′-ATAGAAATAACCAGACGTAAGTT-3′) for *rpoD* gene amplification (Yamamoto et al. 2000; Cladera et al. 2004). The all gene amplification was performed in 25 µl of the reaction mixture containing 1 µM of primers, 0.2 µM of each dNTP, 4 mM MgCl_2_, 1U *Taq* DNA Hot Start polymerase (Promega) and 1 µl of DNA template. PCR was performed in a thermal cycler (Biometra) with an initial denaturation (96 °C for 2 min) followed by 35 cycles of amplification (denaturation at 96 °C for 15 s, annealing at 58 °C for 16S rRNA and 50 °C for *rpoD* and *gyrB* for 15 s, extension at 72 °C for 1 min 30 s for 16S rRNA and 45 s for *rpoD* and *gyrB*) and the single final extension (72 °C for 5 min). A 2-µl aliquot of amplified PCR product was separated by gel electrophoresis in 1 % agarose with ethidium bromide in TBE buffer for 30 min and viewed under UV light. The 1500, 786 and 849 bp-long products of 16S rRNA, *rpoD* and *gyrB*, respectively, were purified using the QIAquick PCR Purification Kit (Qiagen) and sequenced by Genomed (Warszawa, Poland).

Multiple-sequence alignments of 16S rRNA, *rpoD* and *gyrB* sequences were constructed with CLUSTAL W2 software, and maximum likelihood trees were constructed with the neighbour-joining technique (Saitou and Nei 1987) using MEGA v. 6.0 software subjected to BLAST analysis (Tamura et al. 2013) with those deposited in the GenBank in NCBI database (http://www.blast.ncbi.nlm.nih.gov). Bootstrap tests using 1000 replicates were performed to test the robustness of each phylogeny (Felsenstein 1985).

### Growth room pot experiment

The soil mixture containing sand and perlite (1:1, w/w) was sterilized in an autoclave under pressure for 30 min during two consecutive days, and the mixture was placed in 9 × 9 cm plastic pots. The seeds of *M.truncatula* Gaertn. Jemalong J5 ecotype (provided by INRA, Versailles, France) were sterilized and scarified using 95 % H_2_SO_4_ for 10 min, rinsed thoroughly with sterile distilled water, placed on water-saturated Whatman No. 1 disks in Petri dishes, and incubated at 4 °C for 4 days. Subsequently, the seeds were incubated at 20 °C for 24 h and the seedlings were planted in the pots. The seedlings were grown for 1 week under controlled growth room conditions with light–dark and temperature cycles (16 h light at 24 °C; 8 h dark at 20 °C). The light density was 150 μmol m^−2^ s^−1^ (Green Power LED modules, Philips). The one-week-old seedlings were inoculated with 10 ml bacteria inoculum (density of 10^8^ CFU ml^−1^) or 10 mM MgSO_4_. The inoculum of non-symbiotic and symbiotic bacteria was prepared by growing bacterial cells in 20 ml of TSB and 2xYT medium, respectively, and incubated in a shaker incubator (200 rpm) at 28 °C. The density of each culture was measured in a Shimadzu UV–Vis 1800 spectrophotometer at 600 nm. The medium was subsequently separated from the culture by centrifugation (8000*g*/10 min/4 °C). The cells were suspended in 20 ml of sterile 10 mM MgSO_4_. Following centrifugation (8000*g*/10 min/4 °C), the supernatant was discarded and the washing procedure was repeated twice. The cell suspension was diluted fivefold by the addition of sterile 10 mM MgSO_4_, and 10 ml portions of the dilution were used for seedling inoculation with a pipette, at a distance of 1 cm from the stem. The plants were irrigated with distilled water. The soil was fertilized once a week with N-low fertilizer (pH 5.8) containing (mg l^−1^): KNO_3_ (13), KH_2_PO_4_ (17), CaCl_2_ × H_2_O (46), MgSO_4_ × 7H_2_O (15), K_2_SO_4_ (27), EDTA_2_Na_2_Fe (5) and microtraces (µg l^−1^) of: H_3_BO_3_ (371), MnSO_4_ × H_2_O (209), KCl (337), ZnSO_4_ × 7H_2_O (33), (NH_4_)_6_Mo_7_O_24_ × 4H_2_O (33), CuSO_4_ × 5H_2_O (17), H_2_SO_4_ (17). The shoot, root fresh and dry (dried at 105 °C for 24 h) weights were determined 4 weeks after inoculation.

### Plant growth-promoting traits

All the strains used in *M. truncatula* growth experiments were analyzed for their ability to produce indolic compounds, their ACCD activity, siderophore, ammonia and organic acid production as well as for their ability to solubilize phosphate and zinc compounds. Additionally, the *phl*D gene detection in pseudomonads was analyzed. In addition, the IAA content in leaves after 3 and 7 days from inoculation of 5-week-old seedlings with selected strains was determined.

#### Bacterial indolic compounds production

The indole compounds quantification was performed with Salkowski’s reagent (Khalid et al. 2004). The non-symbiotic and symbiotic bacteria were cultured overnight in 10 ml of TSB and 2xYT medium, respectively, in a shaker incubator (200 rpm) in the dark at 28 °C. Subsequently, 20 µl aliquots were transferred to 8 ml of DF or M9 salts minimal medium supplemented with l-tryptophan (500 µg ml^−1^). The density of each culture was measured spectrophotometrically at 600 nm (ca. 5 × 10^8^ CFU ml^−1^). Subsequently, the bacterial cells were removed from the culture medium by centrifugation (8000*g*/10 min). A 1-ml aliquot of the supernatant was mixed vigorously with 4 ml of Salkowski’s reagent (150 ml of concentrated H_2_SO_4_, 250 ml of distilled water, 7.5 ml of 0.5 M FeCl_3_ × 6H_2_O) and incubated at 20 °C for 20 min. Indole production was measured spectrophotometrically using absorbance at 535 nm. The concentration of indolic compounds in each culture medium was determined by comparison with a standard curve plotted for pure IAA (Sigma-Aldrich) and reported as µg IAA ml^−1^ of bacterial culture (density ca. 5 × 10^8^ CFU ml^−1^).

#### ACC deaminase activity

The ACCD activity was measured by a modified ACCD activity assay following Penrose and Glick (2003) and Honma and Shimomura (1978). The bacterial cells were incubated overnight in 20 ml of TSB or 2xYT medium in a shaker incubator (200 rpm) at 28 °C. After incubation, the bacterial culture was centrifuged (8000*g*/10 min/4 °C). The supernatant was removed and the cells were washed with 10 ml DF or M9 salts minimal medium, and centrifuged again. The cells were suspended in 7.5 ml DF or M9 salts minimal medium with 5 mM ACC. The bacterial cells were incubated at 28 °C for 24 h. The culture was centrifuged (8000*g*/10 min/4 °C), the supernatant was removed, and the cells were washed with 0.1 M Tris–HCl (pH 7.6). The washing procedure was repeated twice. A 1-ml bacterial suspension was centrifuged at 16,000*g* for 5 min and the supernatant was removed. The pellet was suspended in 600 µl 0.1 M Tris–HCl (pH 8.0) to which 300 µl toluene were added, and the cell suspension was vortexed for 30 s. A 100-µl aliquot of protein extract was kept from each sample to determine protein concentration of each sample using the Bradford method (Bradford 1976). Toluenized cells (200 µl) were placed in microcentrifuge tubes with 20 µl of 0.5 M ACC and incubated at 30 °C for 15 min. Following the addition of 1 ml of 0.56 M HCl and mixing, the cell suspension was centrifuged (16,000*g*/5 min/RT). Subsequently, 800 µl of 0.56 M HCl was added to 1 ml of the supernatant and the mixture was vortexed. The dinitrophenylhydrazine reagent (0.2 % 2,4-DNP in 2 M HCl) in the amount of 300 µl was added to the glass tube and incubated at 30 °C for 30 min. Before the measurement, 2 ml of 2 N NaOH was added and the absorbance was measured at 540 nm. The ACCD activity was determined by measuring the production of α-ketobutyrate and comparing the result with a standard curve using an α-ketobutyrate (Honma and Shimomura 1978). The enzyme activity was expressed as micromoles of α-KB mg protein^−1^ h^−1^.

#### PCR analysis with specific *phl*D primers

Bacterial DNA was used for PCR amplification with specific *phl*D gene primers (Phl2a and Phl2b) designed by Raaijmakers et al. (1997). *Phl*D gene amplification was performed in 25 µl reaction mixture containing 1 µM of each primer, 0.2 µM of DNA template each dNTP, 4 mM MgCl_2_, 1U *Tag* Hot Start polymerase (Promega) and 1 µl of DNA template. This reaction mixture was incubated in a thermal cycler (Biometra) with initial denaturation (96 °C for 2 min) and then cycled 35 times through the following temperature profile: denaturation at 96 °C for 15 s, annealing at 60 °C for 15 s, extension at 72 °C for 1 min, and the single final extension at 72 °C for 5 min. A 2-µl aliquot of the amplified PCR product was separated by gel electrophoresis in 1 % agarose with ethidium bromide in TBE buffer for 30 min and was visualized with a UV transilluminator. The 720-bp PCR product was sequenced by Genomed. The phylogenetic tree was inferred by the neighbour-joining method using MEGA v 6.0.

#### Phosphate and zinc compounds solubilization

The solubilization was detected by the formation of transparent halos (mm) surrounding bacterial colonies on the Pikovskaya medium containing insoluble CaHO_13_P_3_ or ZnO after 10 days at 28 °C (Pikovskaya 1948).

#### Medium acidification

To detect the ability of the bacterial strains to acidify medium, the isolates were spotted on the TSA medium (pH 7.5) with bromothymol blue, which allowed to detect acidification (DuPree and Wilcox 1977). The medium surrounding the organic acid-producing strains changed colour from blue to green as pH dropped below 7.

#### Siderophore production

The siderophore production was detected by the production of orange halos surrounding the bacterial colonies on a standard Chrome Azurol-S (CAS) agar plates after 72 h at 28 °C (Schwyn and Neilands 1987; Alexander and Zuberer 1991).

#### Ammonia production

The ammonia production by the bacterial strains was determined after their incubation in peptone water for 72 h at 28 °C (Cappuccino and Sherman 1992). Nessler’s reagent (0.5 ml) was added to each tube. The development of brown to yellow colour was a positive test for the ammonia production.

### Protein content determination

The protein content of the enzyme extract was determined by the dye binding method using the Bradford reagent (Sigma-Aldrich) and bovine serum albumin (Sigma-Aldrich) as standard (Bradford 1976).

### IAA determination in seedling leaves by UPLC-MS/MS

Extraction and purification of indole-3-acetic acid (IAA) was conducted as described by Novák et al. (2012) with minor modifications. Frozen leaves (20 mg FW) of 5-week-old seedlings previously inoculated with bacterial suspension of the strains selected during the study (density of 10^8^ CFU ml^−1^) or treated with 10 mM MgSO_4_ (control) were homogenized using a MixerMill (Retsch GmbH, Haan, Germany) and extracted in 1 ml 50 mM sodium phosphate buffer (pH 7.0) containing 1 % sodium diethyldithiocarbamate and stable isotope-labeled internal standard (5 pmol of ^13^C-IAA per sample). The pH was adjusted to 2.7 with 1 M hydrochloric acid, and the samples were purified by solid phase extraction. The extracts were purified on Oasis HLB columns (30 mg, Waters Corp., Milford, MA, USA), conditioned with 1 ml methanol, 1 ml water and 0.5 ml sodium phosphate buffer (pH 2.7). After sample application, the column was washed with 2 ml 5 % methanol and then eluted with 2 ml 80 % methanol. Eluates were evaporated to dryness and dissolved in 30 µl of mobile phase prior to mass analysis using an Acquity UPLC^®^ System and triple quadrupole mass spectrometer Xevo™ TQ MS (Water) (Floková et al. 2014).

### Statistical analysis

Each experiments was run in five replicates and repeated twice. Analysis of variance (ANOVA), Duncan’s multiple range test and Pearson’s correlation coefficients were calculated using Statistica for Windows v. 9.0 (StatSoft Inc., Tulsa, OK, USA).

## Results

### Isolation and identification of bacterial isolates

Sixteen bacterial isolates were obtained from the rhizospheric soil and root nodule samples from alfalfa. The first step in characterizing and identifying a bacterial culture involved examination of colony morphology and cell shape. After 24-h incubation of isolates on the TSA medium, the size, shape, edge, texture, height, colour and optical properties of bacterial colonies were determined. Most isolates (85 %) formed small (1–2 mm) and medium-sized (3 mm) colonies. Only two isolates: KK 8b and KK 11, formed large colonies, about 4 mm in diameter. Colonies formed by most isolates (75 %) were circular, the remaining 25 % were irregular in shape. All the colonies had smooth edges, their texture being smooth as well. As many as 81 % of the colonies were convex, one (KK 3) was umbonate, one (KK 11) was crater-like, and one (KK 13) was flat. Twelve of the 16 isolates formed creamy colonies, 2 being whitish and 2 yellowish. All the 16 isolates were found to be rod-shaped (data not shown).

The second step in bacteria identification involved determination of physiological and biochemical characteristics of the isolates. Almost all the isolates, except KK 8a, were able to move (Table 1). Seven isolates (KK 1a, KK 1b, KK 2, KK 3, KK 4, KK 9a, KK 11) reacted positively to Gram staining. Most of the isolates tested were able to hydrolyse starch, while only three isolates (KK 6, KK 9a, KK 10) were able to hydrolyse lactose. As few as 4 isolates tested positively in the resistance to streptomycin test. Three isolates (KK 5, KK 7, KK 9b) were able to fluoresce.

**Table 1:** Physiological and biochemical characteristics of isolates from *Medicago sativa* rhizosphere and nodules

Strain code	Motility ability	Gram staining	Starch hydrolysis	Fermentation of lactose	Streptomycin resistance	Fluorescence
KK 1a	+	+	+	–	+	–
KK 1b	+	+	+	–	+	–
KK 2	+	+	+	–	+	–
KK 3	+	+	+	–	–	–
KK 4	+	+	+	–	+	–
KK 5	+	–	+++	–	–	+
KK 6	+	–	++	+	–	–
KK 7	+	–	+++	–	–	+
KK 8a	–	–	++	–	–	–
KK 8b	+	–	–	–	–	–
KK 9a	+	+	++	+	–	–
KK 9b	+	–	–	–	–	+
KK 10	+	–	–	+	–	–
KK 11	+	+	+	–	–	–
KK 12	+	–	–	–	–	–
KK 13^a^	+	–	–	–	–	–

Reaction index: −, negative; +, low; ++, medium; +++, high

^a^Isolated from nodules

**Fig. 1:**
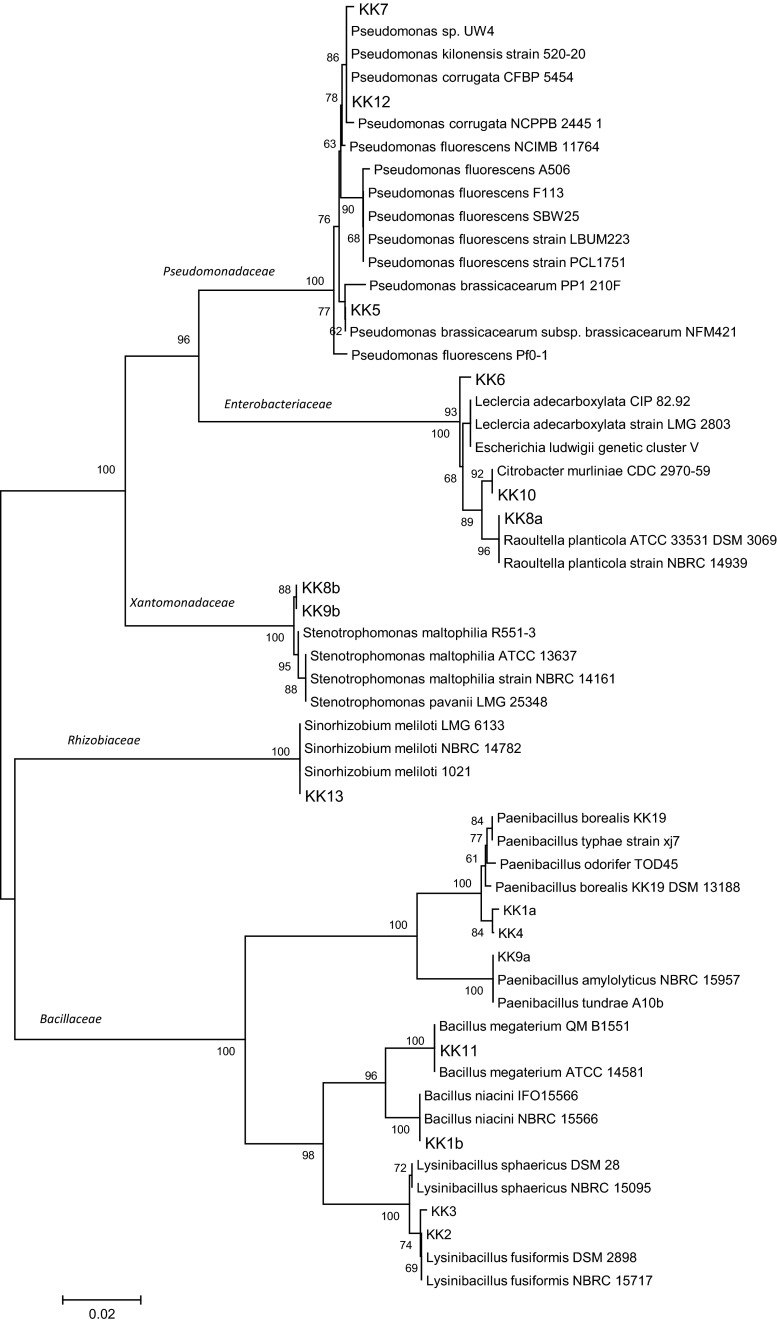
16S rRNA gene sequence-based phylogenetic tree (not rooted) showing position of 16 isolates from *Medicago sativa* rhizosphere and nodules in relation to taxonomically similar bacteria. The analysis was conducted using the neighbor-joining test. The *scale bar* indicates 0.02 changes/site. Bootstraps of 1000 replicates were used and are shown at the branch nodes of the phylogenetic tree

**Fig. 2:**
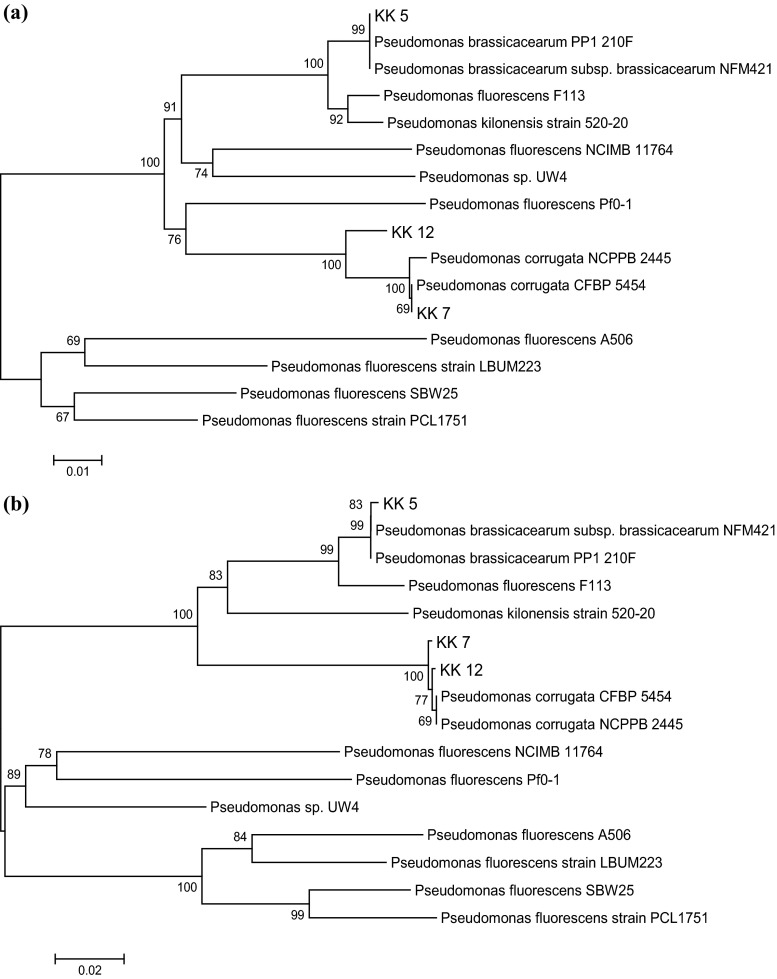
Phylogenetic tree (not rooted) based on *rpo*D (**a**) and *gyr*B (**b**) gene sequence showing position of 3 *Pseudomonas* isolates from *Medicago sativa* rhizosphere in relation to taxonomically similar bacteria. The analysis was conducted using the neighbor-joining test. The *scale bar* indicates 0.01 (**a**) and 0.02 (**b**) changes/site. Bootstraps of 1000 replicates were used and are shown at the branch nodes of the phylogenetic tree

Based on the morphological, physiological and biochemical characteristics, only the isolates KK 1a, KK 1b, KK 2, KK 3, KK 4, KK 9a could be assigned to the families Bacillaceae and Listeriaceae, whereas 9 isolates (KK 5, KK 6, KK 7, KK 8a, KK 8b, KK 9b, KK 10, KK 12, KK 13) were placed in the families Pseudomonadaceae, Enterobacteriaceae, Moraxellaceae, Xantomonadaceae, Burkholderiaceae and Rhizobiaceae. 16S rRNA gene sequencing was used to fine-tune the identification of these bacteria. An about 1.5 kb fragment of 16S rRNA of all the isolates was amplified by PCR products. The PCR products were purified, cloned and sequenced. Nucleotide sequences of the isolates were compared with sequences available in NCBI GenBank. The percentage of 16S rRNA gene sequence similarities (97.7–99.8 %) of all the isolates to the closest type strains is shown in Table 2. The data allowed also to create a dendrogram with 5 separate taxonomic groups (Fig. 1). Seven of the 16 isolates isolated from the *M. sativa* rhizosphere and nodules were assigned to the family Bacillaceae, one to Rhizobiaceae, two to Xantomonadaceae, three to Enterobacteriaceae and three to Pseudomonadaceae. The family Bacillaceae was represented by 3 genera: *Bacillus* (strains KK 1b, KK 11), *Lysinibacillus* strains (KK 2, KK 3) and *Paenibacillus* strains (KK 1a, KK 4, KK 9a) (Table 2; Fig. 1). All strains identified as representing the family Bacillaceae are Gram-positive, the remaining 9 strains being Gram-negative. Only one isolate, KK 13, obtained from the *M. sativa* nodules belongs to the family Rhizobiaceae and the genus *Sinorhizobium* (Table 2; Fig. 1). The next two Gram-negative isolates, KK 8b and KK 9b, obtained from the *M. sativa* rhizosphere belong to the family Xantomonadaceae and the genus *Stenotrophomonas* (Table 2; Fig. 1). The further 3 Gram-negative bacterial isolates, KK 8a, KK 10 and KK 6 represent 3 genus of the family Enterobacteriaceae*: Citrobacter*, *Leclercia* and *Raoultella* (Table 2; Fig. 1). Finally, the family Pseudomonadaceae is represented by 3 isolates (KK 5, KK 7 and KK 12) identified as representing the genus *Pseudomonas* (Table 2; Fig. 1). The use of house-keeping genes *rpoD* (Fig. 2a) and *gyrB* (Fig. 2b) in the taxonomic study of the 3 *Pseudomonas* strains confirmed that the strains KK 5, KK 7 and KK 12 belong to the species listed in Table 2.

**Table 2:** 16S rRNA gene sequencing-based identification of bacteria isolated from *Medicago sativa* rhizosphere and nodules

Strain code	Families	Genus	Closest relative (GenBank no.)	Similarity %	GenBank accession no.
KK 1b	*Bacillaceae*	*Bacillus*	*B. niacini* (AB021194)	99.7	KP858911
KK 11			*B. megaterium* (GU252112)	98.8	KP858923
KK 2		*Lysinibacillus*	*L. fusiformis* (AJ310083)	99.8	KP858912
KK 3			*L. fusiformis* (AJ310083)	99.7	KP858913
KK 1a		*Paenibacillus*	*P. odorifer* (AJ223990)	97.8	KP858910
KK 4			*P. borealis* (AJ011322)	98.3	KP858914
KK 9a			*P. amylolyticus* (AB073190)	97.7	KP858920
KK 13	*Rhizobiaceae*	*Sinorhizobium*	*S. meliloti* (AL591688)	98.8	KP858909
KK 8b	*Xantomonadaceae*	*Stenotrophomonas*	*S. maltophilia* (AB294553)	99.2	KP858919
KK 9b			*S. maltophilia* (CP001111)	98.6	KP858921
KK 10	*Enterobacteriaceae*	*Citrobacter*	*C. murliniae* (AF025369)	99.4	KP858922
KK 6		*Leclercia*	*L. adecarboxylata* (JN175338)	98.8	KP858916
KK 8a		*Raoultella*	*R. planticola* (AB680712)	98.9	KP858918
KK 5	*Pseudomonadaceae*	*Pseudomonas*	*P. brassicacearum* (CP002585)	99.8	KP858915
KK 7			*P. corrugata* (HM190230)	99.5	KP858917
KK 12			*P. corrugata* (HM190230)	99.8	KP858924

### Effects of the bacteria identified on *M. truncatula* seedling growth

**Fig. 3:**
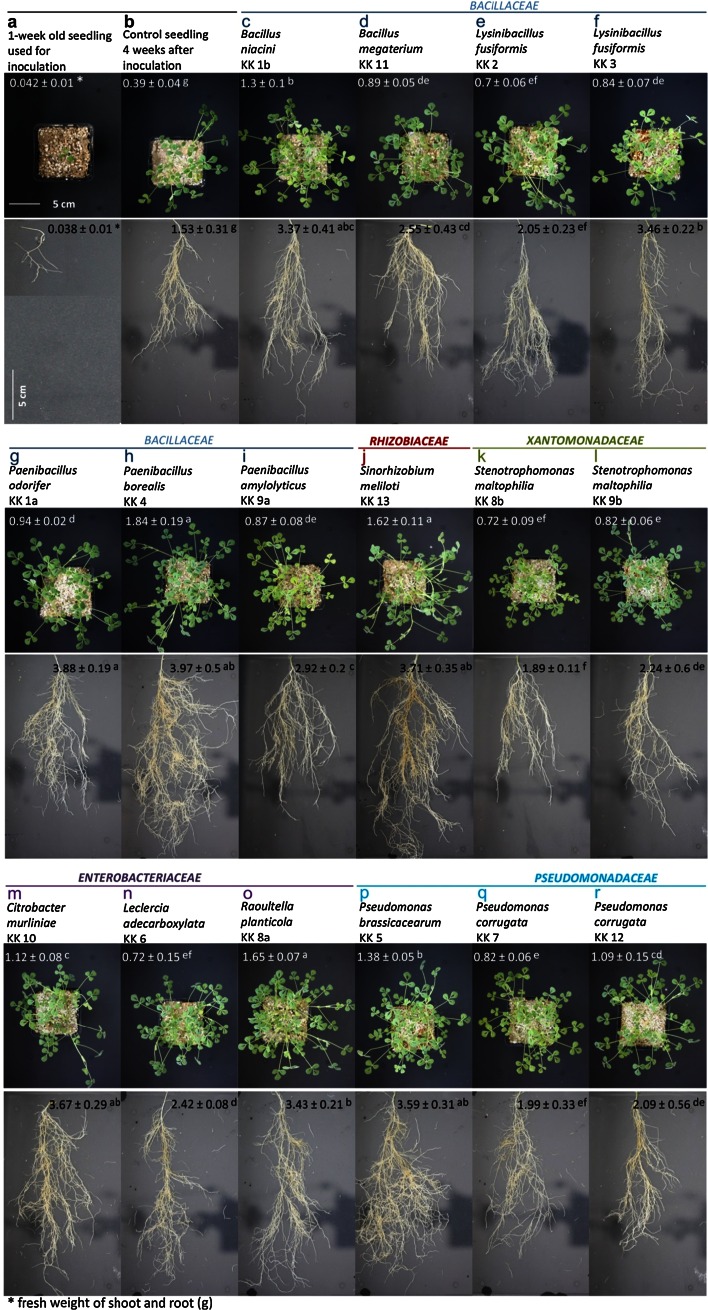
Effects of bacteria isolated from *Medicago sativa* rhizosphere and nodules on *Medicago truncatula* shoot and root growth 4 weeks after inoculation (**c**–**r**). Two-way ANOVA with Duncan’s multiple range test was used to detect significant differences. Means denoted with *different letters* (**a**–**g**) are significantly different (*P* ≤ 0.05)

**Fig. 4:**
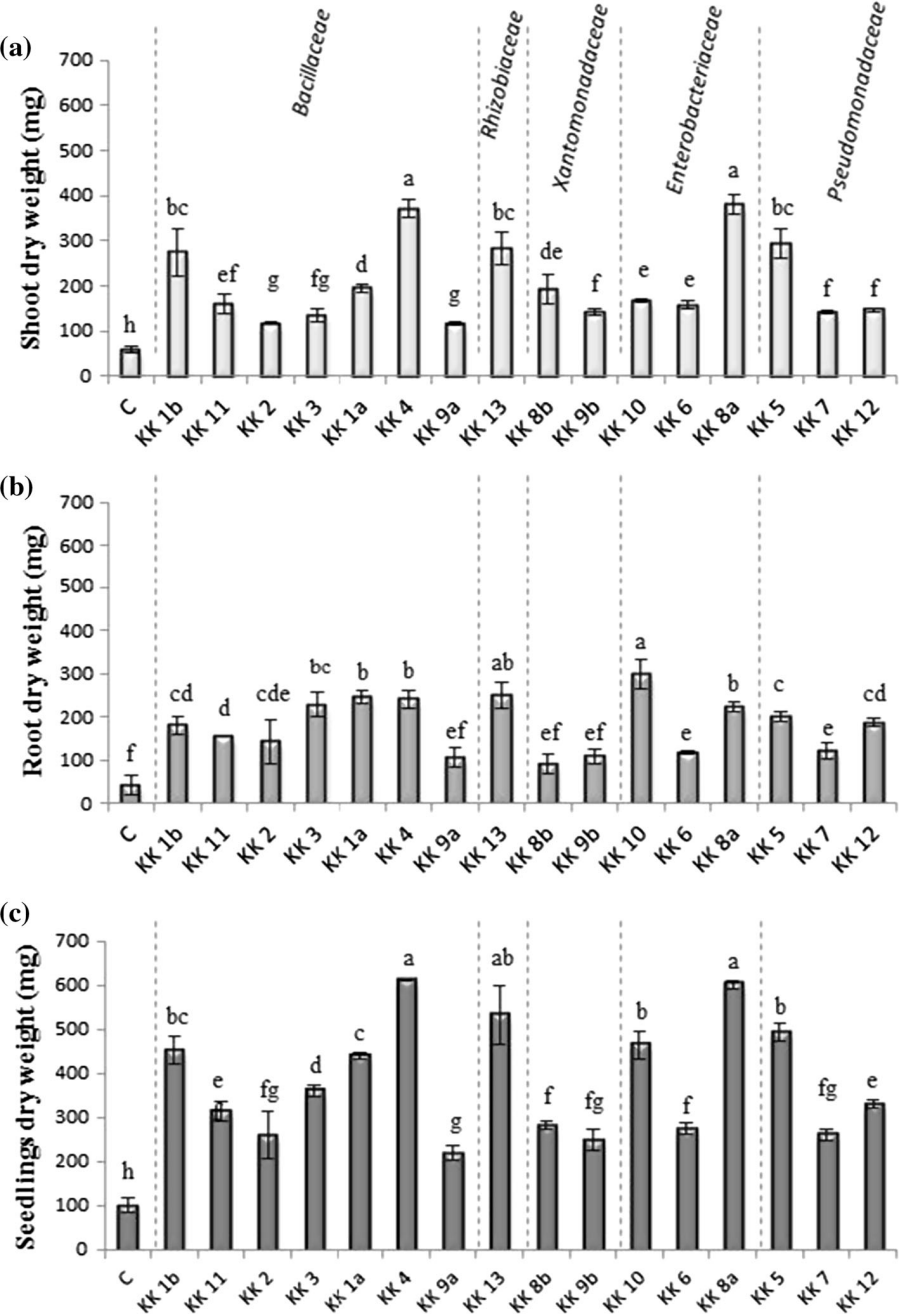
Effects of bacteria isolated from *Medicago sativa* rhizosphere and nodules on dry mass of *Medicago truncatula* shoots (**a**), roots (**b**) and seedlings (**c**) 4 weeks after inoculation. *Vertical bars* indicate ±SD. Two-way ANOVA with Duncan’s multiple range test was used to detect significant differences. Means denoted with *different letters* (**a**–**g**) are significantly different (*P* ≤ 0.05)

Inoculation of 1-week-old *M. truncatula* seedlings (Fig. 3a), grown in soil mixture under controlled conditions, with a suspension of all the strains identified had a positive effect on the seedling development 4 weeks after inoculation (Fig. 3c–r) compared with that of the control (Fig. 3b). Six of the 16 strains showed a particularly high seedling growth promotion ability: *Paenibacillus borealis* KK 4 (Fig. 3h), *Raoultella planticola* KK 8a (Fig. 3o), *Sinorhizobium meliloti* KK 13 (Fig. 3j), *Pseudomonas brassicacearum* KK 5 (Fig. 3p), *Citrobacter murliniae* KK 10 (Fig. 3m) and *Bacillus niacini* KK 1b (Fig. 3c). These bacteria induced a high increase in fresh weight of the roots, 2.2–2.5 times higher than that of the control. Their beneficial effect was also observed in growth of shoots; their fresh weight was almost 3–4.7 times higher that of the control. The bacterial promotional effect on the seedling growth involves not only their impact on the root weight, but also the root architecture. After the seedlings had been inoculated with a suspension of *P. borealis* KK 4 (Fig. 3h) and *P.odorifer* KK 1a (Fig. 3g), they produced the same root fresh weight (ca. 4.0 g), but the architecture of the roots was different. The roots of the seedlings inoculated with *P. borealis* KK 4 were more branched than the roots of seedlings inoculated with *P. odorifer* KK 1a and the fresh shoot weight of the seedlings was twice of that of the latter. Similarly to *P. borealis* KK 4, other bacteria (*R. planticola* KK 8a, *S. meliloti* KK 13, *P. brassicacearum* KK 5, *C. murliniae* KK 10 and *B. niacini* KK 1b) which had a positive impact on the shoot weight increase, distinctly stimulated root branching (Fig. 3o, j, p, m, c). The weakest promotion of root and shoot growth was observed after seedling inoculation with suspensions of KK 8b strain closest relative to *Stenotrophomonas maltophilia*, *Pseudomonas corrugata* KK 7 and *Lysinibacillus fusiformis* KK 2 (Fig. 3k, q, e). Four weeks after inoculation, the seedlings treated with these bacteria showed the least branched roots and the lowest root weight (between 1.89 and 2.05 g), although the weight was still higher than that in the non-inoculated controls. The beneficial effect of inoculation with all the strains was confirmed by data on dry weights of shoots (Fig. 4a), roots (Fig. 4b) and whole seedlings (Fig. 4c) which were all higher than those of the untreated seedlings. The shoot dry weight was the highest in plants inoculated with *R. planticola* KK 8a, *P. borealis* KK 4, *P. brassicacearum* KK 5, *S. meliloti* KK 13 and *B. niacini* KK 1b, which were 6.5, 6.3, 5.0, 4.8 and 4.6 times higher, respectively, than in control plants (Fig. 4a). Inoculation with the remaining 11 strains also substantially (from about 2.0–3.0 times) increased the shoot dry weight, compared to the control. The strain *C. murliniae* KK 10 was the most effective promoter of root biomass (Fig. 4b). The isolate increased the root dry weight 7 times (299 mg), compared with the control (43 mg). The strains *S. meliloti* KK 13, *Paenibacillus odorifer* KK 1a, *P. borealis* KK 4, *L. fusiformis* KK 3 and *R. planticola* KK 8a produced also a marked positive effect on the root dry weight, compared with the non-inoculated control seedlings; the root weight of the treated seedlings was more than 5 times higher (223–250 mg) than that of controls. Four weeks after inoculation with all the strains, whole seedling biomass was several times higher than that of the non-inoculated plants (Fig. 4c). The most effective strains were again *P. borealis* KK 4, *R. planticola* KK 8a, *S. meliloti* KK 13, *P. brassicacearum* KK 5, *C. murliniae* KK 10 and *B. niacini* KK 1b; the seedling dry weights 6.0, 5.8, 5.2, 4.8, 4.6 and 4.5 times higher than those of the control, respectively.

### Bacterial indolic compounds production, ACC deaminase activity and the presence of *phl*D gene

We also evaluated the ability of the strains showing a phytostimulation effect on *M. truncatula* seedlings to produce indolic compounds and examined them with respect to the ACCD responsible for modulation of the rhizosphere ethylene content. All the strains were capable of indolic compounds biosynthesis in the presence of l-tryptophan, the amount produced being found to range from about 4–47 µg per 1 ml of suspension with a density of 5 × 10^8^ cells ml^−1^ (Table 3). The highest amount of these compounds produced resulted from effects of *S. meliloti* KK 13 (47.2 µg ml^−1^), *P. corrugata* KK 12 (46.8 µg ml^−1^), *P. borealis* KK 4 (41.1 µg ml^−1^) and *R. planticola* KK 8a (41.1 µg ml^−1^); the lowest amounts being induced by *P. brassicacearum* KK 5 (4.0 µg ml^−1^). The ability to produce indolic compounds depends not only on a species, but also on a strain; *P. brassicacearum* (KK 5) produced the lowest amount of these compounds among all the strains tested. However, even different strains within a species differed in their ability to produce the indolic compounds: while *P. corrugata* KK 7 produced 24.8 µg ml^−1^, *P. corrugata* KK 12 produced almost twice that, 46.8 µg ml^−1^ (Table 3). The same observation was made when the production of these compounds by three species of *Paenibacillus* was analyzed; *P. amylolyticus* KK 9a, *P. odorifer* KK 1a and *P. borealis* KK 4 produced 29.7, 34.5 and 41.1 µg ml^−1^, respectively. On the other hand, very similar amounts of indolic compounds were synthesized by both *Bacillus* species: *B. niacini* KK 1b and *B. megaterium* KK 11 produced 37.1 and 36.8 µg ml^−1^, respectively.

**Table 3:** Indolic compounds production and ACC deaminase activity in bacteria isolated from *Medicago sativa* rhizosphere and nodules

Families	Strain name	Indolic compounds (µg IAA ml^−1^)	ACCD (µmol α-KB mg protein ^−1^h^−1^)
*Bacillaceae*	*Bacillus niacini* KK 1b	37.1 ± 2.6^bc^	0.6 ± 0.01^h^
	*Bacillus megaterium* KK 11	36.8 ± 3.3^bc^	345.3 ± 49.2^b^
	*Lysinibacillus fusiformis* KK 2	32.9 ± 2.4^d^	3.1 ± 0.1^g^
	*Lysinibacillus fusiformis* KK 3	25.4 ± 0.1^e^	3.0 ± 0.07^g^
	*Paenibacillus odorifer* KK 1a	34.5 ± 2.3^cd^	0.7 ± 0.04^h^
	*Paenibacillus borealis* KK 4	41.1 ± 3.0^b^	35.3 ± 0.5^d^
	*Paenibacillus amylolyticus* KK 9a	29.7 ± 4.9^cd^	21.6 ± 0.9^e^
*Rhizobiaceae*	*Sinorhizobium meliloti* KK 13	47.2 ± 2.8^a^	n.d.
*Xantomonadaceae*	*Stenotrophomonas maltophilia* KK 8b	35.4 ± 0.8^c^	5.6 ± 0.1^f^
	*Stenotrophomonas maltophilia* KK 9b	23.2 ± 1.6^ef^	n.d.
*Enterobacteriaceae*	*Citrobacter murliniae* KK 10	30.1 ± 3.8^cd^	3.3 ± 0.04^g^
	*Leclercia adecarboxylata* KK 6	25.7 ± 2.1^de^	0.5 ± 0.01^h^
	*Raoultella planticola* KK 8a	41.1 ± 2.0^b^	0.4 ± 0.01^h^
*Pseudomonadaceae*	*Pseudomonas brassicacearum* KK 5	4.0 ± 0.1^g^	1469.2 ± 136.6^a^
	*Pseudomonas corrugata* KK 7	24.8 ± 1.0^e^	108.2 ± 2.5^c^
	*Pseudomonas corrugata* KK 12	46.8 ± 1.9^a^	34.6 ± 1.2^d^

Two-way ANOVA with Duncan’s multiple range test was used to detect significant differences. Means denoted with different letters (a–h) are significantly different (*P* ≤ 0.05)

*n.d.* not detected

Of the 16 strains, 14 were able to grow on the ACC-containing medium as the sole nitrogen source (Table 3). These strains, representing 13 species, expressed the ACCD activity at levels ranging from 0.4 to about 1469.2 µmol α-KB mg protein^−1^ h^−1^. The highest ACCD activity (1469.2 µmol α-KB mg protein^−1^ h^−1^) was exhibited by *P. brassicacearum* KK 5, followed by *B. megaterium* KK 11 (345.3 µmol α-KB mg protein^−1^ h^−1^) and *P. corrugata* KK 7 (108.2 µmol α-KB mg protein^−1^ h^−1^). The ACCD activity in 8 strains was very low. Those strains include all the three strains representing the Enterobacteriaceae, 4 from the Bacillaceae and one from the Xantomonadaceae. Two strains: *P. amylolyticus* KK 9a and *S. meliloti* KK 13 showed no ACCD activity.

The analyses described above were conducted to assess the potential of the strains to be used as phytostimulants. The seedlings inoculated with strains which did not produce ACCD, but differed in their ability to produce phenolic compounds showed a different reaction in terms of the root and shoot growth. *S. meliloti* KK 13 producing 2 times higher amounts of indolic compounds (47.2 µg ml^−1^), compared to *S. maltophilia* KK 9b (23.2 µg ml^−1^; Table 3) induced also a nearly twofold increase in the fresh weight of both roots and shoots (Fig. 3j, l) as well as in their dry weights (Fig. 4a, b). The whole seedling weight doubled (the seedling dry weight was 2.4 times that of the seedlings treated with KK 9b) (Fig. 4c). Similarly, a beneficial effect on the seedlings growth involving the amount of indolic compounds produced by the bacteria was observed in two Enterobacteriaceae bacteria strains: *R. planticola* KK 8a and KK 6 closest relative to *Leclercia adecarboxylata.* To summarize, the ability of almost the all strains (except for *P. brassicacearum* KK 5) to enhance root growth (dry weight) was positively correlated with the indolic compounds production (*r* = 0.69; *P* = 0.0001) (data not shown).

The ACCD activity in *R. planticola* KK 8a and KK 6 closest relative to *L. adecarboxylata* was at a similar, very low, level (0.42 and 0.45 µmol α-KB mg protein^−1^ h^−1^), but KK 8a showed an about 1.6 times higher ability to produce indolic compounds (41.1 µg ml^−1^) than strain KK 6 (25.7 µg ml^−1^; Table 3), and induced an about 1.4 and 1.9 times higher fresh weight of roots and shoots, respectively (Fig. 3o, n) the dry weight of roots, shoots, and whole seedlings being higher by the factor of 1.9, 2.4 and 2.2, respectively (Fig. 4a–c). A very interesting effect on seedling growth was observed after inoculation with bacteria from two different species of the genus *Paenibacillus*: *P. odorifer* KK 1a (Fig. 3g) and *P. borealis* KK 4 (Fig. 3h). The amounts of indolic compounds produced by those bacteria did not differ much, but the strains differed strongly in their ACCD activity. In *P. borealis* KK 4, the ACCD activity was 52 times higher that of *P. odorifer* KK 1a (Table 3). However, the difference had no effect on the root weight (the fresh and dry weights were almost identical; Figs. 3g, h, 4b), but did affect the root architecture. Roots of the seedlings inoculated with *P. borealis* KK 4 (Fig. 3h) were more branched. Also the shoot fresh weight was twice that of the shoot weight of seedlings pre-treated with *P. odorifer* KK 1 (Fig. 3g). An identical pattern was observed in the shoot dry weight (Fig. 4a). Generally, the ACCD activity did not correlated with the root dry weight (data not shown).

A very surprising result of seedling growth promotion by *P. brassicacearum* KK 5 (Fig. 3p) was observed with respect of the strain’s ability to produce indolic compounds and its ACCD activity. Among all the 16 strains examined, the bacteria in question showed the lowest capacity for indolic compounds production (4.0 µg ml^−1^) and the highest ACCD activity (1469.2 µmol α-KB mg protein^−1^ h^−1^) (Table 3), but the 4-week-old seedlings inoculated with the bacterium showed a well-developed root architecture (Fig. 3p) and high dry weights of both shoot sand whole seedlings (Fig. 4b, c).

**Fig. 5:**
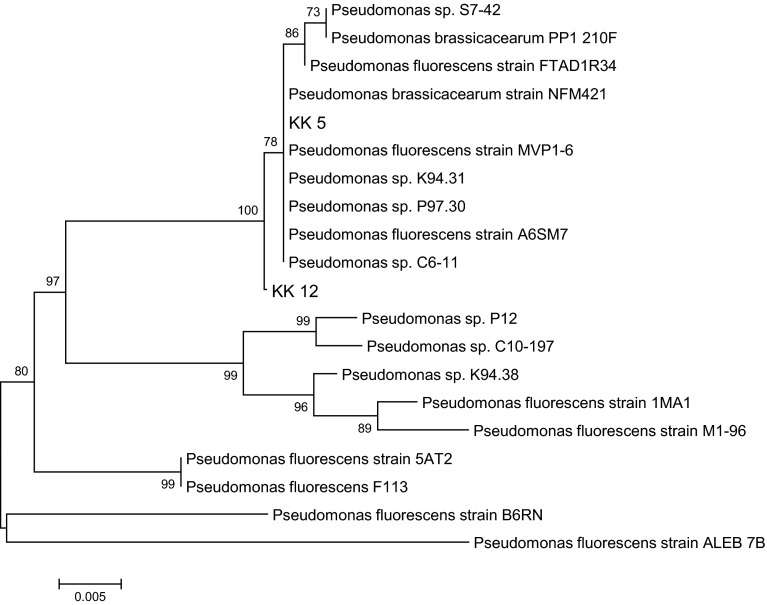
Phylogenetic tree (not rooted) of 2,4-diacetylophloroglucinol biosynthetic protein (PhlD) fragments of two strains (KK 5 and KK 12) inferred by the neighbor-joining method. Bootstraps of 1000 replicates were used and are indicated at the *nodes*. *Scale bar* indicates 0.005 changes/site

To find out whether our three *Pseudomonas* strains contain the *phl*D gene responsible for the synthesis of monoacetylphloroglucinol, the DAPG precursor, PCR amplification of DNAs was performed using primers specifically designed for the *phl*D gene. The presence of the gene fragment was confirmed for two (out of 3) *Pseudomonas* strains: *P. brassicacearum* KK 5 and *P. corrugata* KK 12 (Supplemental Fig. S1). Sequencing of the *phl*D gene fragments from both strains confirmed the expected size of 720 bp. Moreover, as seen in Fig. 5, the similarity to other DAPG precursor genes from fluorescent pseudomonads group is evident.

### Biofertilizing potential of the bacteria

Several tests were used to evaluate the potential of all the isolates to be used as biofertilizers. The ability of all the strains examined to convert insoluble inorganic phosphorus (P) and zinc (Zn) compounds, such as tricalcium phosphate and zinc oxide, to bioavailable forms was studied (Table 4). Six out of the 16 strains were not able to solubilize the insoluble inorganic P compounds. Three strains only (*P. brassicacearum* KK 5, *P. corrugata* KK 7, *R. planticola* KK 8a), when tested in vitro, formed clear halo zones around the colonies kept on Pikovskay’a agar media (high response), while the remaining 7 strains showed a very low P-solubilizing activity. On the other hand, half of the strains tested displayed the ability to dissolve the inorganic zinc compounds. Inorganic zinc oxide was solubilized by all the three Pseudomonadaceae and Enterobacteriaceae strains as well as by one strain each representing the Xantomonadaceae and the Bacillaceae. Thirteen out of 16 strains tested positive for the production of siderophores, iron chelating compounds, as evidenced by the yellow zone on the CAS agar medium. The effect occurred because of iron removal from the blue CAS-FE(III) complex during siderophore production. The most efficient siderophore producers were the following 5 strains*: B. megaterium* KK 11, *P. brassicacearum* KK 5, *P. corrugata* KK 7, KK 8b and KK 6 closest relative to *S. maltophilia* and *L. adecarboxylata*.

**Table 4:** Some biochemical traits of bacteria isolated from *Medicago sativa* rhizosphere and nodules

Families	Strain name	P-sol	Zn-sol	Siderophore production	NH_3_ production	Acidification of medium
*Bacillaceae*	*Bacillus niacini* KK 1b	–	+	–	+	–
	*Bacillus megaterium* KK 11	+	–	+++	++	–
	*Lysinibacillus fusiformis* KK 2	+	–	–	++	–
	*Lysinibacillus fusiformis* KK 3	–	–	+	+++	+
	*Paenibacillus odorifer* KK 1a	+	–	–	+	–
	*Paenibacillus borealis* KK 4	+	–	+	+	–
	*Paenibacillus amylolyticus* KK 9a	+	–	++	+	–
*Rhizobiaceae*	*Sinorhizobium meliloti* KK 13	+	–	+	++	–
*Xantomonadaceae*	*Stenotrophomonas maltophilia* KK 8b	–	–	+++	+	+
	*Stenotrophomonas maltophilia* KK 9b	–	+	++	+	+
*Enterobacteriaceae*	*Citrobacter murliniae* KK 10	–	+++	++	++	–
	*Leclercia adecarboxylata* KK 6	–	+	+++	++	–
	*Raoultella planticola* KK 8a	++	+	+	+	+
*Pseudomonadaceae*	*Pseudomonas brassicacearum* KK 5	+++	+++	+++	++	+
	*Pseudomonas corrugata* KK 7	++	++	+++	++	+
	*Pseudomonas corrugata* KK 12	+	+	++	+	–

Reaction index: −, negative; +, low; ++, medium; +++, high

Ability for produce ammonia (NH_3_) was a trait common to all the 16 strains (Table 4). Nessler’s reaction used to detect ammonia emission by bacteria growing on peptone media showed the highest ammonia amount to have been produced by *L. fusiformis* KK 3.

Six isolates (*L. fusiformis* KK 2, *S. maltophilia* KK 8b and KK 9b, *R. planticola* KK 8a, *P. brassicacearum* KK 5, *P. corrugata* KK 7) showed the ability to acidify TSA medium (Table 4).

### Inoculation with selected rhizobacteria increased the IAA content in *M. truncatula* leaves

**Fig. 6:**
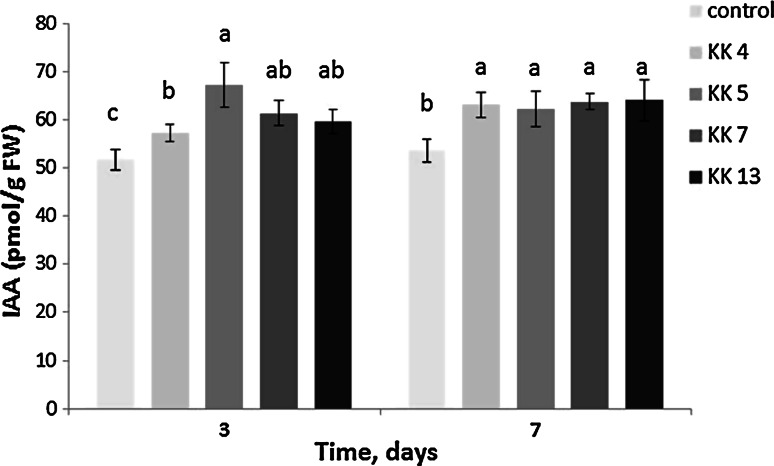
IAA content in leaves of *M. truncatula* seedlings 3 and 7 days after soil inoculation of 5-week-old seedlings with *Paenibacillus borealis* KK 4, *Pseudomonas brassicacearum* KK 5, *P. corrugata* KK 7, *Sinorhizobium melilotii* KK 13. IAA content in leaves of 5-week-old non-inoculated control seedlings at T = 0 was 52.68 ± 3.24 pmol/g FW

To investigate whether the inoculation with bacteria isolated from the rhizospere and nodules of *M. sativa* affects the IAA content in *M. truncatula*, we assayed IAA in leaves of inoculated and non-inoculated plants. For this experiment, we selected four strains. Three of them (*P. borealis* KK 4, *S. meliloti* KK 13 and *P. brassicacearum* KK 5) proved to be the most effective promoters of *M. truncatula* seedling development, whereas one (*P. corrugata* KK 7) showed the weakest ability to promote the root and shoot growth (Fig. 3h, j, p, q). When selecting the strains as representative pseudomonads, their ability to produce indolic compounds, the ACCD activity and the presence of the *phl*D gene were taken into account (Table 3; Supplemental Fig. S1). The IAA amount in leaves of 5-week-old seedlings 3 days after soil inoculation with all the bacterial strains (Fig. 6) was observed to have increased. The highest increase was recorded after inoculation with *P. brassicacearum* KK 5, characterized by the lowest capacity for the production of indolic compounds and the highest ACCD activity (Table 3), and in which the *phl*D gene was detected (Supplemental Fig. S1). On the other hand, *S. meliloti* KK 13, the most efficient producer of indolic compounds but lacking the ACCD activity also increased the endogenous IAA content in the leaves, but the increase was lower than that induced by *P. brassicacearum* KK 5. After 7 days from inoculation of seedlings with all the strains discussed above, the endogenous IAA level in leaves was similar and higher than that in leaves of non-inoculated plants.

## Discussion

Beneficial effects of PGPR on plant growth and yield of many agricultural crops have been shown by numerous studies (Hayat et al. 2010; Bhattacharyya and Jha 2012; Glick 2012; Vacheron et al. 2013). However, to date no data have been published on the positive effects of PGPR isolated from the rhizosphere of *M. sativa* (alfalfa) and their application to promote the development of seedlings of *M. truncatula* (medic barrel). In this study, we isolated bacterial strains from the alfalfa rhizosphere, identified them and evaluated their potential as PGPR for *M. truncatula.*

Based on morphological, physiological and biochemical characteristics, 7 isolates could be pre-assigned to the families Bacillaceae and Listeriaceae, 9 isolates could be identified as representing the Pseudomonadaceae, Enterobacteriaceae, Moraxellaceae, Xantomonadaceae, Burkholderiaceae and Rhizobiaceae. However, by phylogenetic analysis of the 16S rRNA gene sequences it was found that the strains represented 5 rather than 8 families (Bacillaceae, Pseudomonadaceae, Enterobacteriaceae, Xantomonadaceae and Rhizobiaceae). Most strains (7) isolated from the alfalfa rhizosphere belonged to the Bacillaceae. Three strains each were assigned to the Pseudomonadaceae and Enterobacteriaceae, two to the Xantomonadaceae and one to the Rhizobiaceae. The Bacillaceae and Pseudomonadaceae species are common rhizospheric bacteria, and have been isolated from the rhizosphere of different agricultural crops, although there are few data on bacteria isolated from the Fabaceae rhizosphere. The literature contains data on the presence of the Bacilleaceae bacteria [*B. megaterium* strain B153-2-2, *Bacillus fusiformis* (PM-5)] in the soybean rhizosphere (Liu and Sinclair 1992; Park et al. 2005).

The species dealt with in this work have not been reported to be associated with the alfalfa rhizosphere before. Although there is admittedly some information about bacteria isolated from the alfalfa rhizosphere, the authors did not identify the bacteria isolates, but provided codes only (Bharucha et al. 2013a). Similarly, Bharucha et al. (2013b) reported a bacterium identified from the alfalfa rhizosphere, but provided the genus identification only (*Bacillus* sp.). Earlier, Guiñazú et al. (2010) also reported the isolation of bacteria from the alfalfa rhizosphere, but for molecular identification of these isolates they used a too short DNA region (318 bp) for precise taxonomic identification. The authors found the presence of strains assigned to the genera *Pseudomonas* and *Bacillus.*

The present work demonstrated, for the first time, that all the bacteria from the *M. sativa* rhizosphere identified could be effective in enhancing the *M. truncatula* seedling development in soil (sand and perlite mixture) under controlled growth room conditions. The main effects observed 4 weeks after inoculation of 1-week-old seedlings involved an increase in root fresh and dry weights as well as root branching.

A positive effect on shoot development was observed as well. Our results are in line with findings of many other workers who assessed effects of inoculation with non-pathogenic plant growth promoting bacteria (PGPR) on growth of many other plants (Glick 1995, 2012; Wissuwa et al. 2009; Martinez-Viveros et al. 2010; Rashid et al. 2011). Seven out of the 16 bacteria species examined proved to be most effective promoters of the *M. truncatula* seedling development. In terms of the seedling fresh and dry weight, those bacteria may be arranged in the following order: *P. borealis* KK 4 (Bacillaceae), *R. planticola* KK 8a (Enterobacteriaceae), *S. meliloti* KK 13 (Rhizobiaceae), *P. brassicacearum* KK 5 (Pseudomonadaceae), *C. murliniae* KK 10 (Enterobacteriaceae), *B. niacini* KK 1b (Bacillaceae) and *P. odorifer* KK 1a (Bacillaceae).

The approach used in this study included screening of the isolated bacteria for their in vitro indolic compounds biosynthesis and ACCD activity. The production of phytohormones, including auxins, by PGPR is now considered to be one of the most important direct mechanisms by which many rhizobacteria promote plant growth (Spaepen et al. 2007; Spaepen and Vanderleyden 2011; Glick 2012). Among indolic compounds, IAA is mainly known as an inducer of lateral and adventitious root formation (Casimiro et al. 2001; Sorin et al. 2005). Indolic compounds were produced, in the presence of its precursor l-tryptophan (500 µg ml^−1^), by all the 16 isolates from the *M. sativa* rhizosphere. Production of these compounds was quantified as ranging from 4.0 to 47.2 µg ml^−1^. In the presence of tryptophan (1 %), bacterial strains representing three genera: *Pseudomonas*, *Bacillus* and *Azospirillum* produced IAA in amounts ranging from 1.2 to 44.4 µg ml^−1^, but the production was negatively correlated with the root length, while correlating positively with the number of roots in 15-day-old wheat seedlings (Hussain and Hasnain 2011). Our results may suggest that indolic compounds production by bacterial strains, except that by *P. brassicacearum* KK 5, can be responsible for stimulation of *M. truncatula* root development; the indolic compounds content was positively correlated with the root dry mass (*r* = 0.69; *P* = 0.0001)*. P. borealis* KK 4, *R. planticola* KK 8a, *S. meliloti* KK 13, *C. murliniae* KK 10, *B. niacini* KK 1b and *P. odorifer* KK 1a, which promoted root growth most effectively, are characterized by the highest capacity for indolic compounds production in the presence of tryptophan, the plant IAA precursor. Indolic compounds production by these strains ranged from about 30–47 µg ml^−1^. That indolic compounds are mainly responsible for the stimulation of *M. truncatula* seedling growth can be clearly seen when comparing effects of two bacterial strains: *S. meliloti* KK 13 (Rhizobiaceae) and *S. maltophilia* KK 9b (Xantomonadaceae). Showing no deaminase activity, they differ in the ability to produce indolic compounds; KK 13 which produced 2 times more these compounds (47.2 µg ml^-1^) than KK 9b (23.3 µg ml^−1^) was more effective in stimulating the roots mass and branching, and the shoot and seedling weight. A positive correlation between auxin production and growth promoting activity of diverse PGPR has been also reported in *Brassica juncea*, wheat and soya bean (Asghar et al. 2002; Khalid et al. 2004; Wahyudi et al. 2011).

To date, however, no information about such correlations in *M. truncatula* under in vivo conditions is available. Earlier Nolan et al. (2003) showed that when *M. truncatula* leaf explant tissues were cultured in vitro in an auxin-containing medium, they produced numerous roots. However, our study did not always demonstrate such a positive correlation between high levels of bacterial indolic compounds and root development. When the *M. truncatula* seedlings were inoculated with *P. brassicacearum* KK 5, it proved markedly effective in promoting root system development (particularly the lateral roots), but was one of the lowest indolic compounds producers (as little as 4 µg ml^−1^). The promotional activity of this bacteria on the root architecture resulted from a mechanism other than the bacterial indolic compounds. Some strains of fluorescent pseudomonads are known to produce a secondary metabolite, 2,4-diacetylphloroglucinol (DAPG) (Dwivedi and Johri 2003). DAPG can be a signal molecule for plants, as enhancement of root branching in tomato and wheat seedlings has been reported (Brazelton et al. 2008; Couillerot et al. 2011). Application of exogenous DAPG can alter the root architecture of tomato seedlings by interacting with an auxin-dependent signaling pathway (Brazelton et al. 2008; Couillerot et al. 2011). These authors observed also that roots of the auxin-resistant diageotropica tomato mutant showed a reduced DAPG sensitivity with regard to the inhibition of the primary root growth and induction of root branching.

In the present study, we used electrophoresis of the PCR products amplified from DNA of three *Pseudomonas* strains with primers for the *phl*D gene responsible for the synthesis of monoacethylphloroglucinol (MAPG), the DAPG precursor. The detection of the *phl*D gene in *P. brassicacearum* KK 5, and also in *P. corrugata* KK 12, could suggest DAPG production by these bacteria and probably their potential contribution to the observed strong stimulation of root branching in *M. truncatula* seedlings. However, to verify this hypothesis, DAPG quantification has to be performed, and phlD mutants should be generated.

The PGPR which do not produce auxins are also known for their ability to modify the endogenous plant IAA transport or for regulation of auxin homeostasis by, e.g., production of volatile organic compounds (VOC), resulting in the same root architecture effects (Zhang et al. 2007; Contesto et al. 2010; Zamioudis et al. 2013). Our results suggest that endogenous plant IAA, the content of which in seedling leaves increased after 3 and 7 days following soil inoculation with rhizobacteria, may be probably involved in *M. truncatula* seedling growth promotion. Thus, we provide evidence that some PGPR strains can trigger an increase of the endogenous auxin content in leaves, the main site of auxin production. Contesto et al. (2010) showed that up-regulation of several genes involved in tryptophan and IAA biosynthesis took place in shoots of 12-day-old *A. thaliana* seedlings inoculated with *Phyllobacterium brassicacearum* STM196. Consistent with the increased expression levels of genes involved in tryptophan biosynthesis, the inoculated shoots contained higher amounts (80 %) of this IAA precursor compared to the non-inoculated shoots. However, the IAA level in *Arabidopsis* shoots remained essentially unchanged upon inoculation with the strain in question.

Numerous studies have suggested that bacterial IAA and ACCD synergistically promote plant growth (Glick et al. 2007; Glick 2012). The main visible effect of root inoculation with ACCD-producing bacteria is the enhancement of plant root elongation. Patten and Glick (2002) suggested that IAA and ACCD work in concert to stimulate root elongation. Exogenous IAA is known to increase transcription and activity of ACC synthase (Peck and Kende 1995), an enzyme which catalyzes the ACC synthesis in peas. The plant ACC stimulates ACCD activity in bacteria (Li and Glick 2001). An activity of 20 nmol α-KB mg protein^−1^ h^−1^ has been reported (Penrose and Glick 2003) to be sufficient for plant growth promotion. In our study the 16 strains for which the ACC-containing selection medium was used as the sole nitrogen source, 14 showed the ACCD activity to range from 0.2 to about 1469.2 µmol α-KB mg protein^−1^ h^−1^. It appears that some of the *M. sativa* rhizosphere strains analyzed in this study could be among the potentially *M. truncatula* growth promoting bacteria. However, our results show that indolic compounds production by most of the strains isolated from the *M. sativa* rhizosphere, rather than the ACCD activity, is responsible mainly for stimulating *M. truncatula* seedling development (roots and shoots). No correlation between the ACCD activity and the root dry mass of roots was observed. In general, the highest *M. truncatula* seedling growth promotion was obtained after inoculation with strains which produced most of the IAA (between 30 and 47 µg ml^−1^), with simultaneous absence or very low activity of ACCD. Previously, we also suggested that IAA production rather than ACCD activity in two *Agrobacterium rhizogenes* strains, 15834 and LBA1334, might be responsible for the stimulation of the grass *Festuca rubra* L. seed germination, seedling emergence and development upon seed inoculation; mainly by increasing growth of lateral roots and more complex architecture of the branching root system (Król et al. 2014). We compared the effects of seed inoculation of the two strains tested with the ability to produce IAA and of exogenous application of IAA at concentrations corresponding to IAA production by these strains. The presence of exogenous IAA during seed treatment produced a stimulatory effect on seedlings growth, but the effect was lower compared to that of a treatment with bacterial strains. Improvement of *F. rubra* L. seedling development, particularly visible in the root architecture, is achieved because the two bacteria strains are able to produce IAA and have active ACCD which probably prevents the root growth-inhibiting levels of ethylene induced by bacterial or exogenous IAA. However, the exact mechanism of growth stimulation by bacteria is still unclear. This is distinctly visible on the example of the *P. brassicacearum* KK 5, a strain that is an excellent promoter of *M. truncatula* seedling growth: the strain exerted a particularly positive effect on the root system development, but was also least able to produce IAA (only 4 µg ml^−1^) among the strains examined, and was characterized by the highest ACCD activity (up to 1467 µmol α-KB mg protein^−1^ h^−1^). However, Contesto et al. (2008) described that the effects of the *acds*-deficient mutants of *Phyllobacterium brassicacearum* STM196, *P. putida* UW4, *Rhizobium leguminosarum* bv*. viciae* 128C53K and *Mesorhizobium loti* MAFF303099 on the root system architecture of *Arabidopsis* seedlings were not significantly different from those of their wild-type counterparts. The only exception found concerned the *acdS* mutant of *P. putida* UW4 which triggered an increase in lateral root number, as opposed to the WT strain which did not affect this number. In turn, *P. brassicacearum* strain Am3 producing ACCD (ca. 10 µmol α-KB mg protein^−1^ h^−1^) showed both pathogenic and growth promoting properties in its interaction with tomato seedlings (Belimov et al. 2007), depending on the density of the inoculant used. At a low bacterial concentration (10^6^ cells ml^−1^), increased in vitro root elongation and root biomass of soil-grown tomato were recorded; however, a higher concentration of the strain mentioned produced a negative effect on root elongation. The ACCD-deficient mutant of strain Am3 (T8) decreased root elongation and biomass production, compared to the wild-type Am3. In our experiments, the density of *P. brassicacearum* KK5 inoculant was high (10^8^ cells ml^−1^) and positive effects on root and seedling development were observed. Similarly, it was found earlier that *P. brassicacearum* Am3 increased rape and pea plant growth in pot experiments when the plants were inoculated with a high density suspension (5 × 10^7^ cells ml^−1^) (Safronova et al. 2006). Thus, it is becoming increasingly apparent that each rhizobacterium can promote plant growth by several mechanisms. In the case of *M. truncatula* seedlings and *P. brassicacearum* KK 5, a low level of bacterial indolic compounds (4 µg ml^−1^), a high ACCD activity (1467 µmol α-KB mg protein^−1^ h^−1^), the presence of *phl*D gene responsible for biosynthesis of the DAPG precursor and an increase in endogenous levels of IAA in leaves may probably be involved in promoting the development of seedlings. There is clear evidence that plant growth promotion by rhizobacteria involves more mechanisms than one.

Moreover, PGPR can additionally help plants by increasing their uptake of nutrient elements such as P and Zn on account of their ability to solubilize the unavailable nutrient forms, which is one of the essential criteria in facilitating the transport of most nutrients, including P and Zn (Wissuwa et al. 2009). The low availability of macronutrient P to plants results from the fact that the vast majority of soil P is in insoluble forms (both organic and inorganic), and plants can only absorb P in two soluble forms: the monobasic ($\text{H}_{2} \text{PO}_{4}^{ - }$) and the diabasic ($\text{HPO}_{4}^{ 2- }$) ions. Strains representing the genera *Pseudomonas*, *Bacillus* and *Rhizobium* are among the most powerful phosphate solubilizers (Richardson et al. 2009; Hayat et al. 2010). In our study, all the three *Pseudomonas* strains were capable of solubilizing the insoluble tri-calcium phosphate [Ca_3_(PO_4_)_2_], *P. brassicacearum* KK 5 showing the highest solubilizing ability. Five of the seven Bacillaceae strains were characterized by a low ability to solubilize this compound in question. Jorquera et al. (2008) isolated P-solubilizing bacteria from the rhizospheres of the cultivated Fabaceae plants such as *Trifolium repens* and *Lupinus luteus*. Cattelan et al. (1999) found only two of five rhizospheric isolates that tested positive for solubilization had actually a positive effect on soybean seedling growth. In earlier studies, phosphate (P)-solubilizing bacteria such as *Bacillus* and *Paenibacillus* sp. were applied to soils to enhance the phosphorus status of plants (Van Veen et al. 1997). Thus, it is likely that many of the so-called biofertilizers exert a dual effect that is mediated by direct solubilization of inorganic P by organic acids synthesized and excreted by soil bacteria, mineralization of organic P carried out by phosphohydrolases such as acid phosphatases, and a stimulatory effect by IAA production (Khan et al. 2009). It has been suggested that PGPR which decrease the medium pH during growth through their ability to produce organic acids are efficient P solubilizers (Nautiyal et al. 2000). Seven out of the 16 isolates from the alfalfa rhizosphere were able to acidify the TSA medium. Three of these were representatives of the *Pseudomonadaceae* (KK 5, KK 7, KK 12), two were members of the Xantomonadaceae (KK 8b, KK 9b) and one each belonged to the Enterobacteriaceae (KK 8a) and Bacillaceae (KK 3).

In addition, some of the bacteria examined can help to increase the micronutrient (Zn) supply by solubilizing inorganic zinc compounds (Wissuwa et al. 2009). Among the strains tested, those representing the Enterobacteriaceae and Pseudomonadaceae showed the highest ZnO dissolving potential.

Iron, essential for cellular growth and metabolism, has—like phosphorus—a low mobility and is not easily available to plants, but may be supplied by bacteria. Many plants are known to use various bacterial siderophores as an iron source, although the total concentrations are probably too low to contribute substantially to plant iron uptake (Martinez-Viveros et al. 2010). Siderophores, as iron Fe-chelating agents, are produced by various types of bacteria including the Gram-negative *Pseudomonas* and Gram-positive *Bacillus* (Bhattacharyya and Jha 2012). In our experiments, all the strains isolated from the alfalfa rhizosphere did produce siderophores which were detected using the chrome azurol S assay, a general test for siderophores. The most positive reaction in this test was shown by the bacteria representing the genera *Pseudomonas*, *Bacillus*, *Xanthomonas* and *Enterobacter.*

Ammonia (NH_3_) produced by rhizobacteria is an additional N source in soil (McNeill and Unkovich 2007). All the bacteria from the *M. sativa* rhizosphere were able to produce NH_3_ under in vitro condition in the presence of peptone. Fourteen strains are also potential ammonia producers because of the presence of ACCD which degrades ACC to ammonia and α-ketobutyrate. When ammonia accumulates in the plant cells at levels higher than 0.1 mM, it can inhibit some processes such as seed germination and seedling establishment, but it can also stimulate root branching. So the beneficial effect of *P. brassicacearum* KK 5 observed is likely to be additionally related to the very high activity of ACCD in ammonia production. All these attributes of the bacteria examined may affect the nutrient uptake abilities of *M. truncatula.*

This study illustrates the significance of screening the rhizobacteria under in vitro conditions for multiple PGPR traits and their evaluation under controlled conditions in pot experiments. The results obtained have led to the selection of effective PGPR isolates representing 5 families (Bacillaceae, Rhizobiaceae, Xantomonadaceae, Enterobacteriaceae, Pseudomonadaceae) which—as a result of their multiple PGPR traits—could prove effective in improving the seedling development of *M. truncatula*, a molecular model crop plant. Screening strategies for selecting the best strains will require more comprehensive knowledge on beneficial effects of the rhizobacteria on growth and development of roots, which is of a critical for effective soil exploration and access to nutrients. *M. truncatula* is a good molecular model which allow root traits to be more readily identified and manipulated, and makes it possible to find molecular markers for specific root traits and identification of genes involved in root development. All the 16 strains identified from the rhizosphere of *M. sativa* and particularly 6 of them (*P. borealis* KK 4, *R. planticola* KK 8a, *S. meliloti* KK 13, *P. brassicacearum* KK 5, *C. murliniae* KK 10 and *B. niacini* KK 1b) appear to be interesting candidates, since inoculations with all these strains was found to affect the root architecture in *M. truncatula.*

Thus, it is becoming increasingly apparent that most PGPR strains can promote plant growth by several mechanisms, although most studies currently focus on individual mechanisms and have not yet been able to sort out the relative contribution of different processes responsible for successful plant growth promotion.

### *Author contribution statement*

EK initiated and designed research, interpreted the results and wrote the manuscript. AK conducted experiments and statistical analysis. Both authors approved the manuscript.

## Electronic supplementary material

Below is the link to the electronic supplementary material.

Supplemental Fig. S1 Electrophoresis of PCR products amplified from DNA of *Pseudomonas* strains with *phl*D primers. Lane M, DNA 1-kb ladder marker; lane 5, *P. brassicacearum* KK 5; lane 7, *P. corrugata* KK 7; lane 12, *P. corrugata* KK 12 (DOCX 27 kb)

## Abbreviations

ACC: 1-Aminocyclopropane-1-carboxylate acid
ACCD: 1-Aminocyclopropane-1-carboxylate deaminase
DAPG: 2,4-Diacetylphloroglucinol
IAA: Indole acetic acid
PGPR: Plant growth promoting rhizobacteria
