# Supplementary figures and images for: Medicago truncatula Gaertn. as a model for understanding the mechanism of growth promotion by bacteria from rhizosphere and nodules of alfalfa
Source: Planta. 2016 Feb 10;243:1169–89. doi: 10.1007/s00425-016-2469-7 (PMC4837224; doi:10.1007/s00425-016-2469-7)

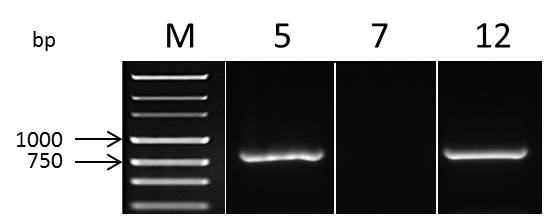


**Suplemental Fig. 1**

Supplement: Supplementary file 1 — Supplemental Fig. S1 Electrophoresis of PCR products amplified from DNA of Pseudomonas strains with phlD primers. Lane M, DNA 1-kb ladder marker; lane 5, P. brassicacearum KK 5; lane 7, P. corrugata KK 7; lane 12, P. corrugata KK 12 (DOCX 27 kb) [file 425_2016_2469_MOESM1_ESM.docx]
